# Registration of transthoracic impedance signal and ventilation volume data in out-of-hospital cardiac arrest

**DOI:** 10.1016/j.dib.2025.111345

**Published:** 2025-02-01

**Authors:** Emma Menant, Bruno Tassart, Céline Meillier, Frédéric Lemoine, Alexandre Petermann, Daniel Jost, Xavier Jouven

**Affiliations:** a Université Paris Cité, INSERM U970, Paris Cardiovascular Research Centre (PARCC), Integrative Epidemiology of Cardiovascular Disease, 56 rue Leblanc, Paris 75015, France; b Emergency Department, Paris Fire Brigade, 1 place Jules Renard, Paris, France; c Laboratoire ICube, UMR 7357, 300 boulevard Sébastien Brant, Illkirch-Graffenstaden, France

**Keywords:** Insufflation, Tidal volume, Cardiopulmonary resuscitation, Cross-correlation

## Abstract

Studying ventilation during out-of-hospital cardiac arrest (OHCA) presents significant challenges due to the limited methods available for monitoring ventilation during Basic Life Support care. Researchers are increasingly focusing on transthoracic impedance (TTI) as a new means of investigating ventilation.

We employed manual ventilation monitoring devices to record cardiopulmonary resuscitation (CPR), ventilation volumes (Vvol) and TTI data. A registration of TTI with Vvol signals is performed. The Vvol are considered as the ground truth for ventilation detection in our dataset. The latter comprises data recorded during OHCA involving adult patients. Specifically, the data include TTI signals and automated external defibrillators (AED) analysis markers collected using Defigard Touch 7® AEDs (Schiller Medical, Wissembourg, France), as well as CPR Vvol recorded by manual ventilation monitoring devices (EOlife®, ARCHEON Medical, Besançon, France).

The TTI signals and Vvol data that derived from the same OHCA can be registered. It allows better characterization of the TTI signal by identifying when TTI variations are caused by ventilations and distinguishing these from artifacts. This registration process allows to position the ventilation on TTI.

The combination of TTI signals and Vvol data improves readability of CPR process, by providing a robust method to interpret TTI signals.

Specifications Table

**Table:** 

Subject	Biomedical engineering
Specific subject area	Collection of ventilation data during cardiopulmonary resuscitation (CPR) in adult out-of-hospital cardiac arrest (OHCA).
Type of data	Raw tables and signals
Data collection	Data recorded by an automated external defibrillator (AEDs) during OHCA in adult patients: transthoracic impedance (TTI) signals and AED analysis markers. The AED used was Defigard Touch 7® (Schiller Medical, Wissembourg, France). Data recorded by a manual ventilation monitoring device placed between the ventilation bag and the mask: ventilation volumes (Vvol). The manual ventilation monitoring device used was EOlife® (ARCHEON Medical, Besançon, France). For each patient, TTI signals, AED analysis markers and Vvol were recorded simultaneously by two CE-marked devices.
Data source location	Institution: Paris Fire Brigade (BSPP: Brigade des Sapeurs-Pompiers de Paris) City: Paris Country: France
Data accessibility	Repository name: Insufflated Volumes and Electrical Impedance for Cardiac Arrest Resuscitations Data identification number: 10.17632/34gffrm87w.1 Direct URL to data: https://data.mendeley.com/datasets/34gffrm87w/1
Related research article	None

## 1 Value of the Data

• We collected two distinct types of data: ventilatory and impedance data. These datasets, along with the method of interpreting both transthoracic impedance (TTI) signals and ventilation volumes (Vvol), offer significant benefits for research on cardiac arrest monitoring.
• The data allow for the identification of precise moments when TTI variations are attributable to ventilation, distinguishing these from artifacts.
• The Vvol serves as a ground truth for detecting ventilation on TTI.
• A time alignment of TTI and Vvol, further called registration, is performed.
• Although initially developed for data from a single manufacturer, we expect the general principles to apply to other manufacturers and datasets.

## 2 Background

Studying ventilation during OHCA is challenging due to the lack of methods to monitor ventilation during Basic Life Support (BLS) care. TTI signals is known to carry information about ventilations [[1], [2], [3]].

In 1965, LH Hamilton et al. showed the correlation between variation of TTI and lung volume change in healthy volunteers [4]. Since ME Valentinuzzi et al. and H Losert et al. found a relationship between TTI variation over tidal volume variation and the weight of animals or mechanically ventilated patients [5,6]. These studies show that CPR ventilations are potentially visible on the TTI signal recorded by the defibrillator during OHCA.

Nevertheless, many factors may affect the visibility of ventilation on TTI, such as: weight or sex, but also pulmonary diseases and other confounding variables for patient outcomes [7,8]. Moreover, care givers can generate TTI variations that can be misinterpreted as ventilations.

A ground truth is needed to study ventilation. A manual ventilation monitoring device was used to record the Vvol during CPR.

## 3 Data Description

**Fig. 1:**
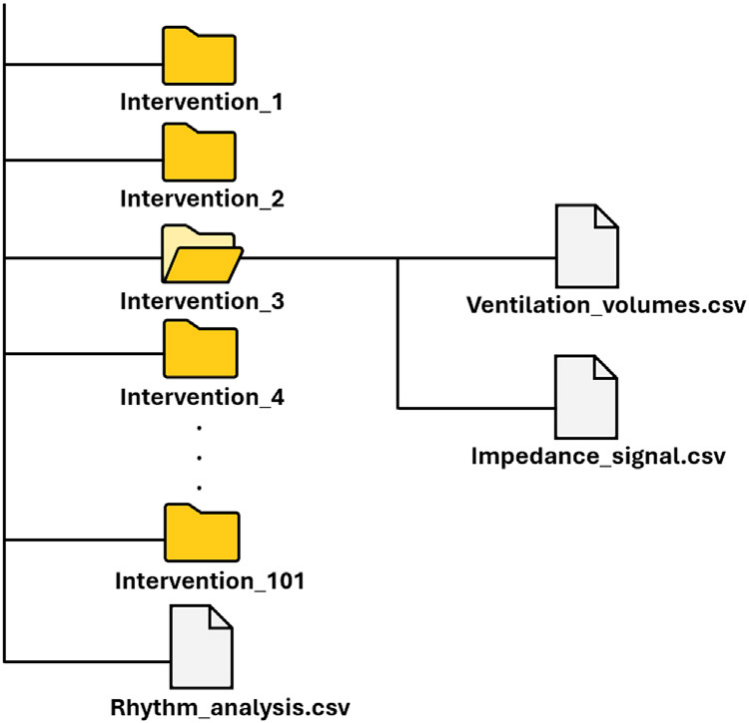
Dataset architecture.

The data consist of two different datasets, each available in table format (Ventilation_volumes.csv and Impedance_signal.csv), and one common table (rhythm_analysis.csv). Thus, in the storage system presented in Fig. 1, each OHCA corresponds to a folder containing two tables. Table 1 summarizes the type and quantity of data available.

**Table 1:** Ventilation_volumes.csv description.

Ventilation volume table (N = 101)	
Cycle number	Nth detected ventilation
Time (s)	Time elapsed since the device power-on, in seconds
Vi (mL)	Insufflated volume, in millilitres
Vt (mL)	Tidal volume, in millilitres (≤ Vi)

**Fig. 2:**
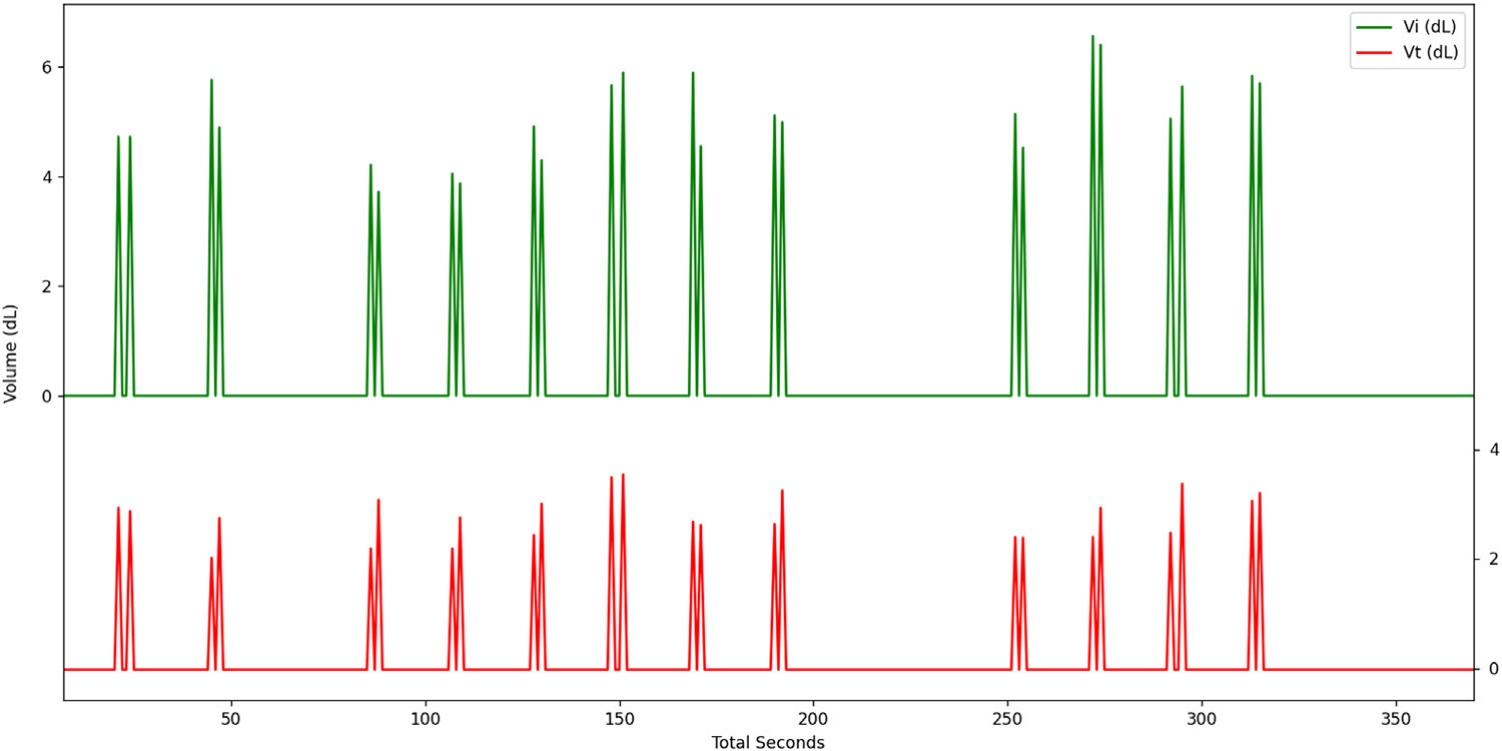
Vvol values display as a signal with a sampling frequency of 1 Hz, insufflated volumes (Vi) in green and tidal volumes (Vt) in red.

The Vvol data are available in “Ventilation_volumes.csv” files. For each cycle, the following measurements are recorded in the format presented in Table 1: the time elapsed since the device power-on (in seconds), the insufflated volume and the tidal volume (in millilitres). Fig. 2 gives an example of Vvol visualization: insufflation volumes are shown in green, and tidal volumes in red.

**Fig. 3:**
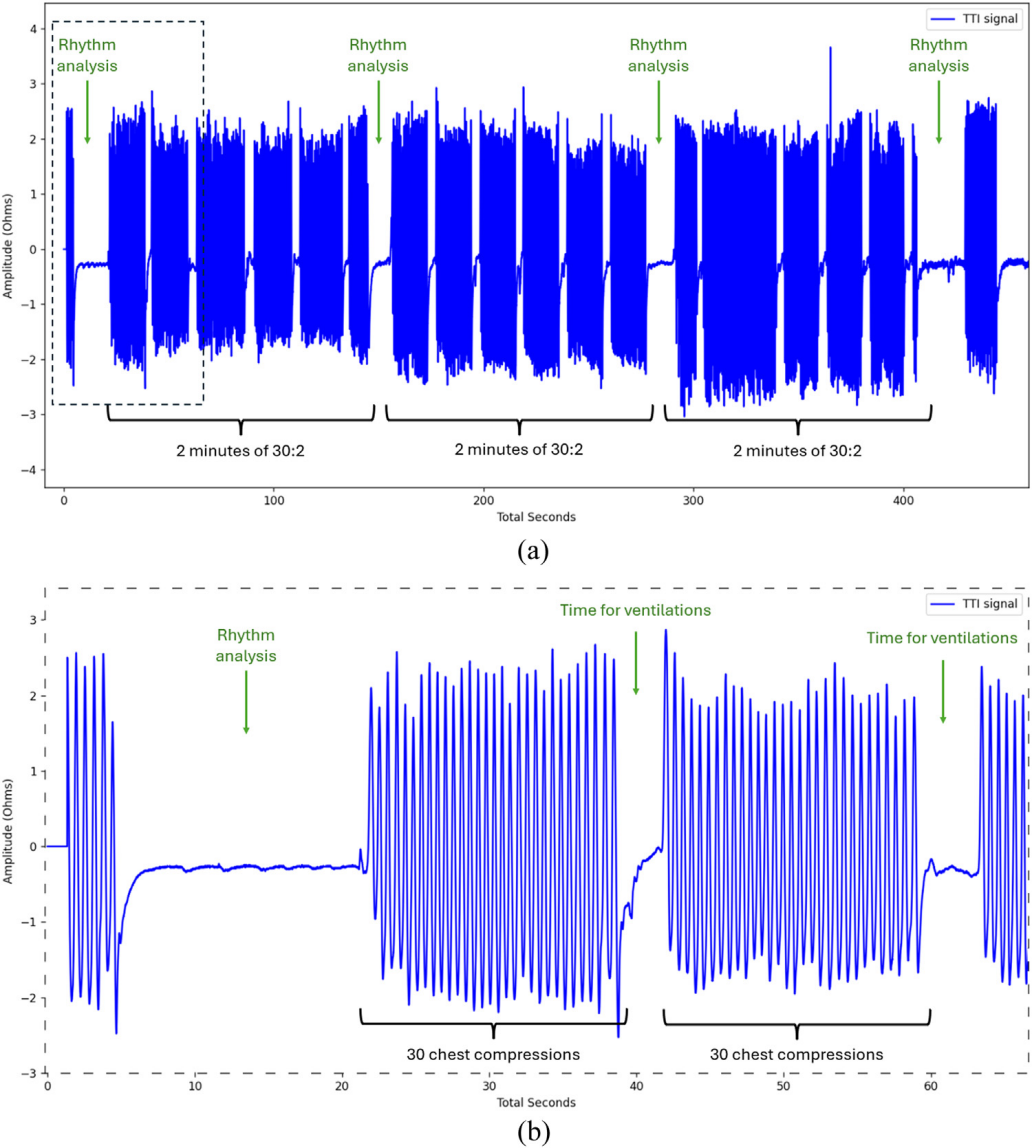
Visualisation of a TTI signal. Panel (B) is a zoom of the dashed area of Panel (A).

The TTI data are available in ``Impedance_signal.csv'' files. The measurements are: time elapsed since the device power-on (in seconds) and the corresponding TTI value (in ohms). Fig. 3 is an example of TTI signal (Table 2).

**Table 2:** Impedance_signal.csv description.

Transthoracic impedance signal table (N = 101)	
Time (s)	Time elapsed since the device power-on, in seconds
Impedance (Ω)	TTI in ohms

The rhythm analysis data are available in the ``rhythm_analysis.csv'' file. Each row represents one OHCA. The data available are the intervention ID and a list of the times of the AED analysis markers (in seconds) (Table 3).

**Table 3:** Rhythm_analysis.csv description.

Rhythm analysis table (N = 1)	
Intervention_id	Number of the intervention
List_analysis (s)	List of the times of the AED analysis markers, in seconds (time elapsed from the device power-on to the analysis start)

## 4 Experimental Design, Materials and Methods

### 4.1 Data acquisition

The BSPP firefighters serve Paris and its suburbs, covering an area of 657 km² and a population of 6 835 500 people (INSEE 2020 [[10], [11], [12], [13]]). The French emergency system is two-tiered physician-manned. In 80 % of adult OHCA cases, firefighter's BLS teams are the first responders. They manage OHCAs according to the current European Resuscitation Council guidelines [9]. ALS teams follow within minutes, usually 10 to 15, and provide medical care on the field.

CPR can be composed of two phases. One phase is characterized by cycles of 30 chest compressions (CC) followed by two ventilations (30:2 CPR). Another phase is continuous CC in parallel with superimposed ventilations. The AED ECG is analysed every 2 min.

The selection criteria for this study were: adult OHCA patients cared for by the BSPP BLS equipped with an AED and a manual ventilation monitoring device from May 2023 to November 2023.

AEDs (Defigard Touch 7®, Schiller Medical SAS, Wissembourg, France) recorded TTI signals and additional events during resuscitation, while manual ventilation monitoring devices (EOlife®, Archeon, Besançon, France) recorded Vvol.

**Fig. 4:**
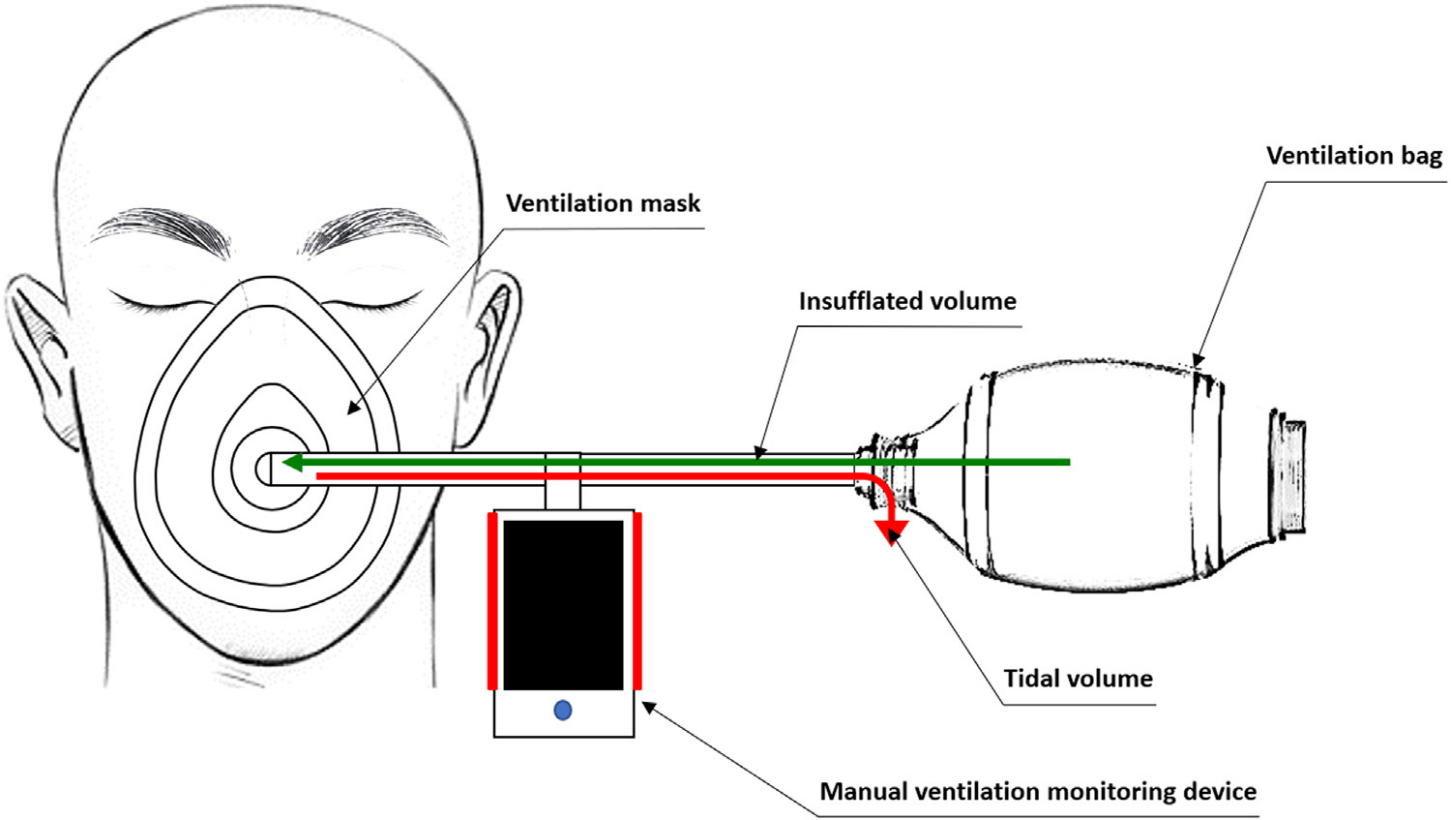
EOlife® medical device.

At the start of resuscitation, the designated rescuer assembles the ventilation equipment in 1 to 2 min, depending on the rescuer and area layout. The monitoring device is placed between the face mask and the resuscitation bag without affecting assembly time (Fig. 4).

The EOlife® ventilation monitoring device measures the incoming and outgoing air volumes through a flow sensor. The EOlife® is specifically designed to monitor BLS ventilations and can record a wide range of ventilation volumes, including small volumes. The data is transmitted via Bluetooth to a tablet provided for this purpose, and then sent in .csv format to a dedicated server.

Nine rescue centres participated in the study, representing 27 emergency vehicles equipped with an EOlife®. In total, 129 OHCA were included in the study. Twenty-eight OHCA have been excluded, 17 because TTI was unavailable and 11 because Vvol was unavailable. Thus, 101 OHCA were eligible for registration.

### 4.2 Registration method

The defibrillator and the manual ventilation monitoring device are not powered on at the same time. Thus, there is a time lag between the TTI signal and the Vvol. In addition, ventilations may not be visible at the same time on TTI and Vvol, as TTI fluctuations correspond to lung inflations and Vvol detections to insufflations.

To find this lag, we used the segments of TTI signal and of Vvol corresponding to the 30:2 CPR periods. Indeed, unlike continuous CC with continuous ventilation, the 30:2 CPR creates a pattern on the Vvol and TTI, and we used these patterns for the registration.

The maximum of the cross-correlation between the binary square wave signal modelling the expected ventilation from TTI signal and the binary signal of detected ventilation allowed us to find the lag.

•Binary square wave signal modelling the expected ventilation from TTI signal

**Fig. 5:**
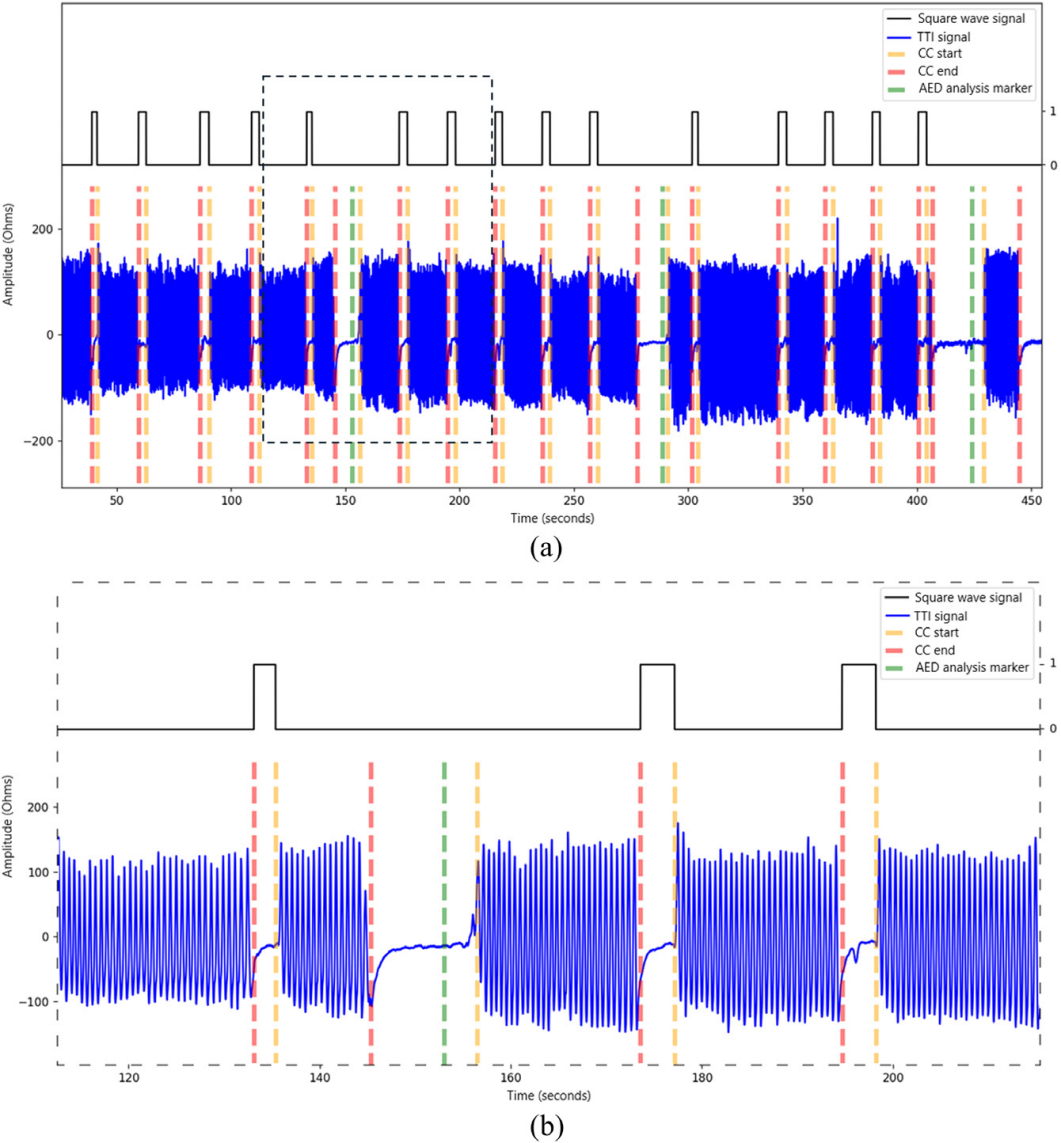
Binary square wave signal modelling the expected ventilation from TTI signal. Panel (B) is a zoom of the dashed area of Panel (A).

The square wave signal is set to 1 between a CC end and a CC start, if there is no AED analysis, else set to 0. The signal value is set to 1 when ventilations are theoretically present. The binary square wave signal is represented in black in Fig. 5.

To create the binary square wave signal modelling the expected ventilation from TTI signal, the CC start and CC end should be annotated. TTI signals are very sensitive to CC [[14], [15], [16], [17]].

An open-source algorithm, developed by Kern and Orlob, can be used to automatically find the CC starts and CC ends [18,19]. As this algorithm was originally developed for accelerometer signals, the TTI signals need to be pre-processed by removing artefacts due to saturation, external electric shock or disconnected electrodes, normalizing the signal and filtering it with a bandpass filter.

•Binary signal of detected ventilation

We created a binary signal of detected ventilations to avoid giving more weight to large ventilation volumes in the computation of cross-correlation (see Eq. (1)).

**Fig. 6:**
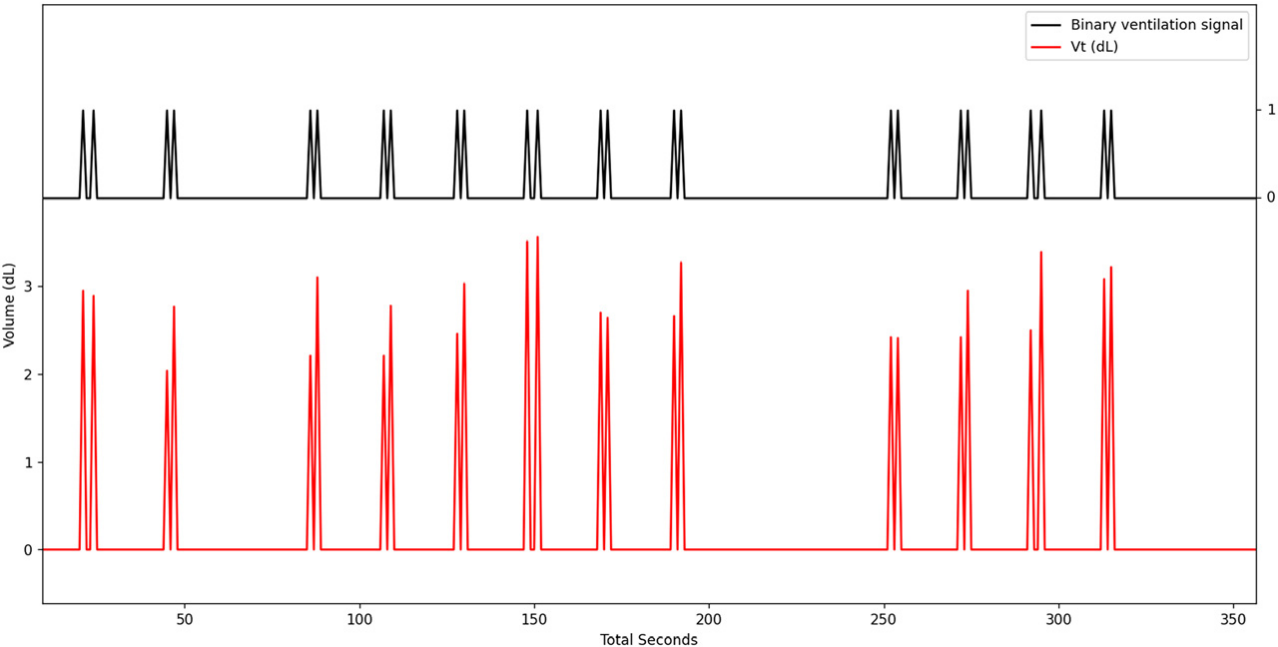
Binary signal of detected ventilation.

The binary signal of detected ventilation is created with the Vt values detected by the manual ventilation monitoring device. The Vt value is used because of its robustness to noise. However, some Vt could not be recorded due to leakage. The Vi was used instead of Vt for OHCA which had not enough Vt values. When a volume is recorded, the binary signal is 1; elsewhere, it is 0 (Fig. 6).

•Cross-correlation function

Cross-correlation is a statistical measure that quantifies the degree to which two signals are correlated at different lags (Eq. (1)). This can reveal whether two signals have similar patterns or trends and at what time intervals these similarities occur.

(1) $$R_{xy}\left[s\right]=\;\sum_{n=-\infty }^{+\infty }x\left[n+s\right]\;y\left[n\right]$$

Eq. (1): Cross correlation formula

The lag s that maximizes the cross correlation $R_{xy}\left[s\right]$ between x the binary signal of detected ventilation and y the binary square wave signal of expected ventilations is the lag between the TTI signal and the Vvol.

**Fig. 7:**
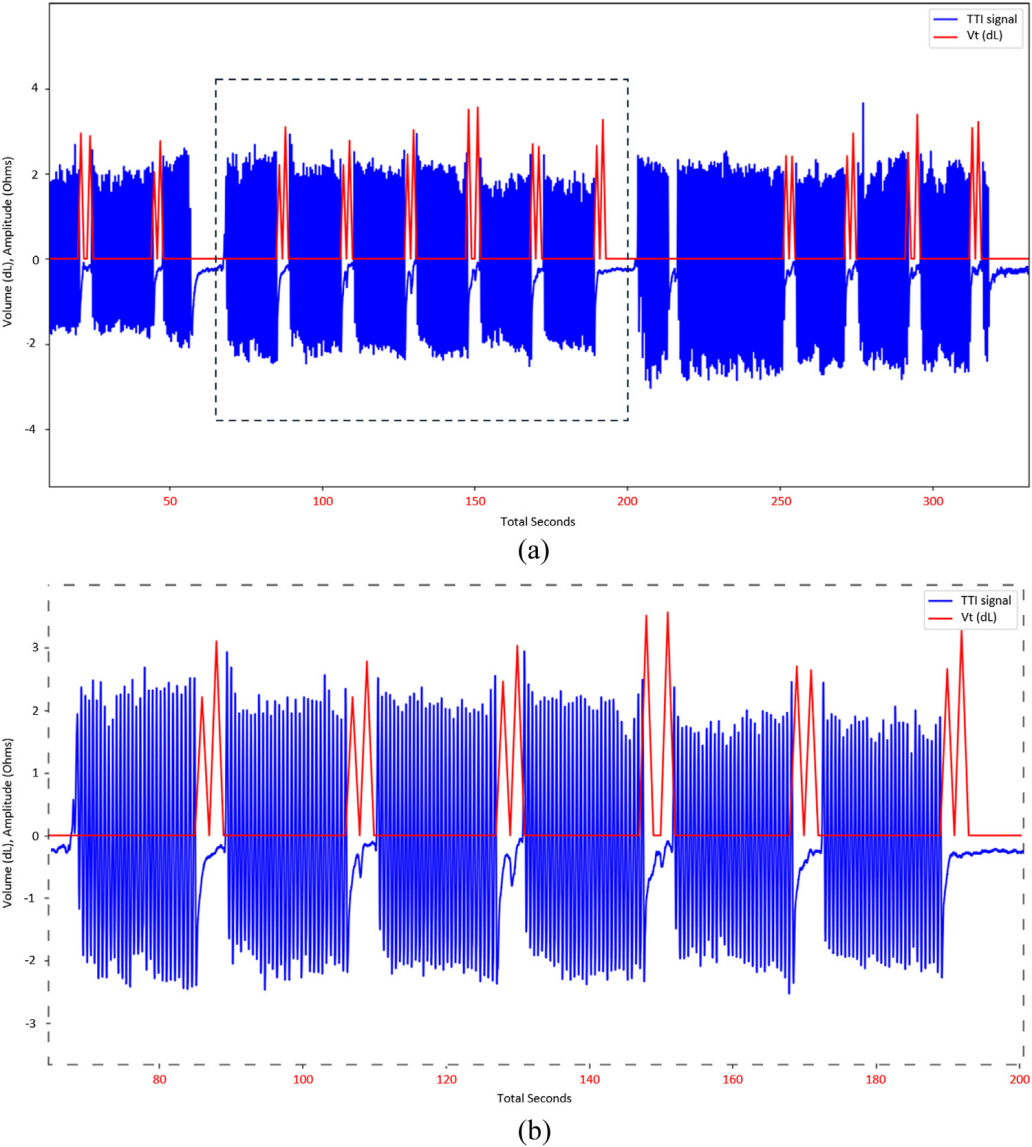
Registration of the Vvol and the TTI with time corresponding to Vvol in red. Panel (B) is a zoom of the dashed area of Panel (A).

Then, we register both TTI and Vvol signals with the determined lag value to check if they match in 30:2 CPR periods and outside (Fig. 7). Two physicians (DJ and XJ) verified the registered signals.

## Limitations

Among 101 OHCA with TTI signal and Vvol data available, 12 (11.9 %) could not be registered. The registration was unsuccessful for 8 OHCA that had no 30:2 CPR period on either TTI signal or Vvol data, 3 OHCA that had less than ten 30:2 CPR cycles, leaving open the possibility that TTI signal and Vvol data may not overlap, and 1 OHCA that had a very low amplitude TTI signal.

Our registration method is based on the 30:2 CPR pattern. Therefore, intercorrelation does not work if firefighters do not comply with 30:2, for example by not ventilating during the dedicated CC pauses.

The registration is impossible if the common part of TTI signal and Vvol data does not include enough 30:2 CPR cycles. The ventilation monitoring device is manually switched on by the rescuers on site after the defibrillator is set up and cardiopulmonary resuscitation has begun (following the completion of priority patient care actions). The defibrillator and ventilation monitoring device are switched off at the end of the resuscitation or upon the arrival of the medical team (ALS team). As AED and EOlife® were not powered on and off simultaneously, the intersection of the parts recorded with each device could be limited.

Another limitation of our work is that the method was tested using two devices from only two manufacturers, which also resulted in a limited amount of data. However, the registration method is designed to be generalizable to other similar devices, provided they include a defibrillator and a ventilation monitoring/measuring device, leaving open the possibility for other researchers to develop larger datasets.

## Ethics Statement

The data comes from a study conducted by the BSPP, in accordance with ethical guidelines and established standards for research involving human subjects. The clinical trial protocol was reviewed and approved by the ethics committee of Ile de France 1 (ref CPP: CPPIDF1-2023-DI19-cat3). The clinical trial registration number is NCT 05992454 and the IDRCB number is 2022-A02771-42.

The patients’ personal information was handled confidentially and anonymized to ensure their privacy. Each patient was individually informed by a letter that their data would be used for research purposes. They could object to this use within one month.

The data collection and analysis were performed in accordance with the principles of the Helsinki Declaration and current regulations regarding the protection of personal data.

The data processing was conducted under the confidentiality conditions defined by the law of January 6, 1978, relating to data processing, files, and freedoms (CNIL), as well as by the General Data Protection Regulation.

## CRediT Author Statement

**Emma Menant:** Conceptualization, Methodology, Software, Validation, Data Curation, Writing - Original Draft, Vizualisation. **Bruno Tassart:** Conceptualization, Methodology, Software, Validation, Data Curation, Writing - Original Draft, Vizualisation. **Céline Meillier:** Writing - Reviewing and Editing, Supervision. **Frédéric Lemoine:** Resources. **Alexandre Petermann:** Resources. **Daniel Jost:** Resources, Writing - Reviewing and Editing, Supervision. **Xavier Jouven:** Writing - Reviewing and Editing, Supervision, Project administration.

## Data Availability

Mendeley DataInsufflated Volumes and Electrical Impedance for Cardiac Arrest Resuscitations (Original data).
